# Balloon Occlusion of the Contralateral Iliac Artery to Assist Recanalization of the Ipsilateral Iliac Artery in Total Aortoiliac Occlusion: A Technical Note

**DOI:** 10.1155/2013/647850

**Published:** 2013-05-15

**Authors:** Abdel Aziz A. Jaffan

**Affiliations:** Division of Interventional Radiology and Image-Guided Medicine, Department of Radiology, Emory University School of Medicine, 1364 Clifton Road NE, Atlanta, GA 30322, USA

## Abstract

Endovascular recanalization of chronic total aortoiliac occlusion is technically challenging. Inability to reenter the true aortic lumen, following retrograde iliac recanalization, is one of the most common causes of failure. We describe a case of a total aortoiliac occlusion where balloon occlusion of the right common iliac artery, following its recanalization from a brachial approach, was used to facilitate antegrade recanalization of the occluded contralateral left common iliac artery.

## 1. Introduction 

Chronic obstruction of the aortic bifurcation, involving to a varying degree and extent both the infrarenal aortic and common iliac arteries (CIAs), can result in a triad of symptoms consisting of intermittent claudication, absent or diminished peripheral pulses, and impotence. Classically described in males, the anatomic pattern of obstruction is equally common in males and females [1]. The onset is mainly between 40 and 60 years of age, and the disease can lead to severe impairment of walking capacity, rest pain, and wheelchair dependence [1, 2]. Aortobifemoral bypass has been considered the gold standard for treating chronic aortoiliac occlusions [3], but results of endovascular recanalization of the iliac arteries now approach those of aortobifemoral bypass with reduced morbidity and mortality and shorter hospitalization [4]. The Trans-Atlantic Inter-Society Consensus for the management of Peripheral Arterial Disease (TASC II) document classifies total aortoiliac occlusion as type D, with surgery as the treatment of choice [3]. Nowadays, endovascular therapy is increasingly performed in patients with extensive aortoiliac disease, including total aortoiliac occlusion [1]. We describe a technique that might facilitate the recanalization of chronic total iliac occlusion, during endovascular recanalization of a total aortoiliac occlusion.

## 2. Case Description 

**Figure 1:**
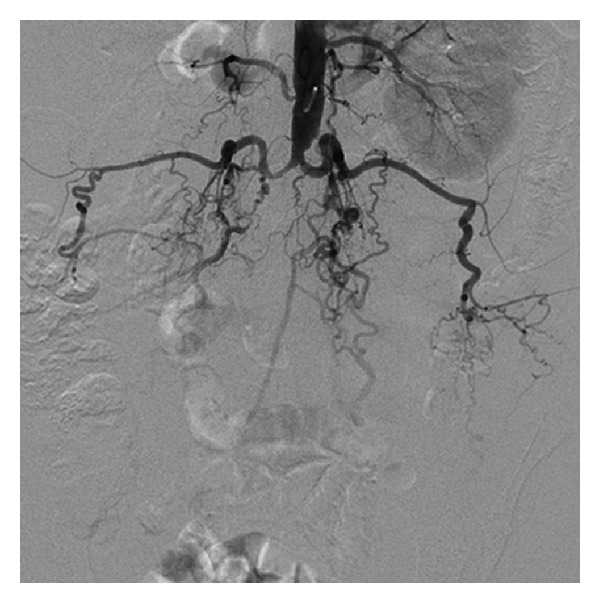
Aortogram showing total occlusion of the distal abdominal aorta and bilateral common iliac arteries.

**Figure 2:**
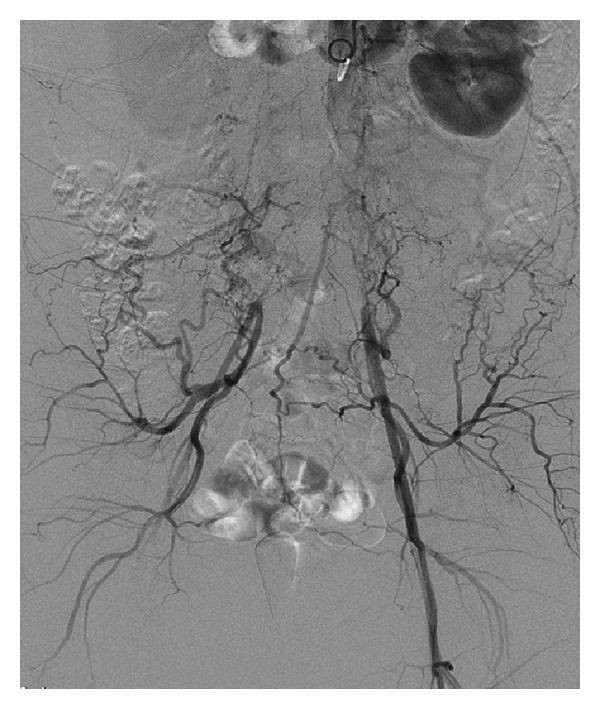
Aortogram showing reconstitution at the level of the iliac bifurcations.

**Figure 3:**
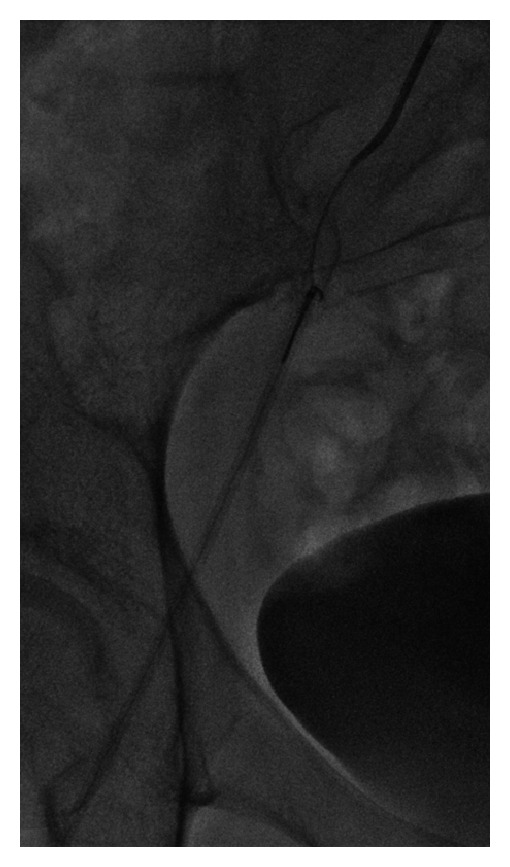
Wire retrieval from the right femoral sheath following recanalization of the right common iliac artery.

**Figure 4:**
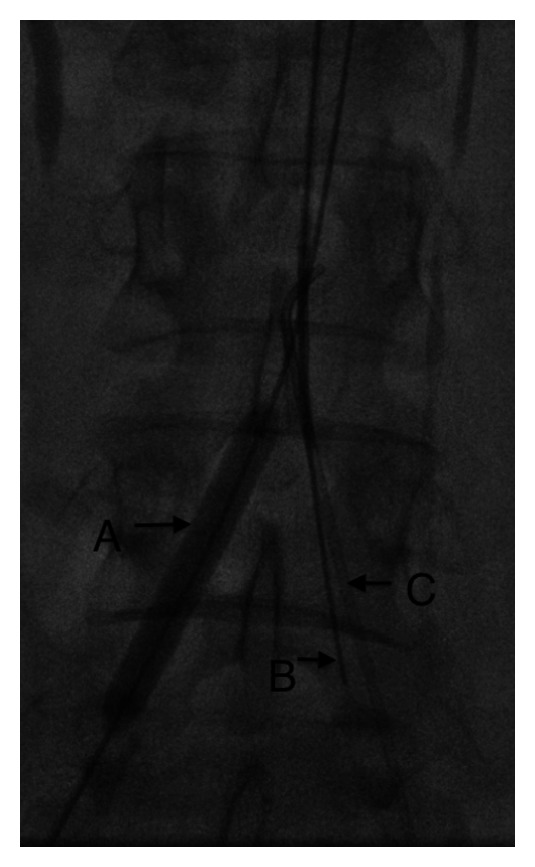
Spot image at the level of the aortic bifurcation showing an inflated angioplasty balloon occluding the recanalized right CIA at its origin (A), a stiff Glidewire advanced into the occluded left CIA from an antegrade brachial approach (B) and a 5 French angiographic catheter in the retrograde subintimal tract of the left CIA advanced from a ipsilateral femoral approach.

**Figure 5:**
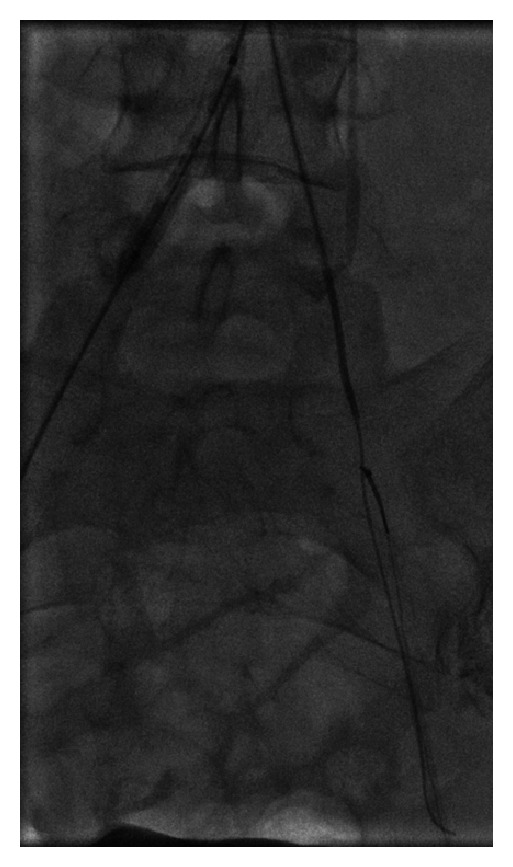
Wire retrieval from the left femoral sheath following recanalization of the left common iliac artery.

**Figure 6:**
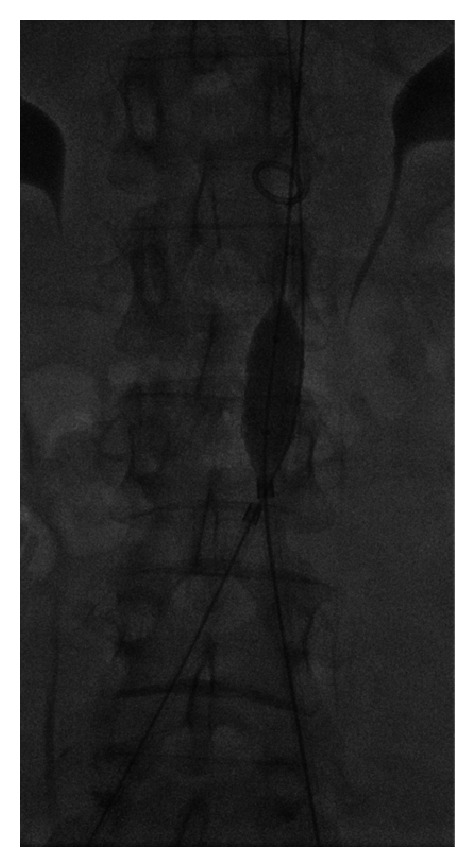
Deployment of the balloon expandable stent in the distal aorta.

**Figure 7:**
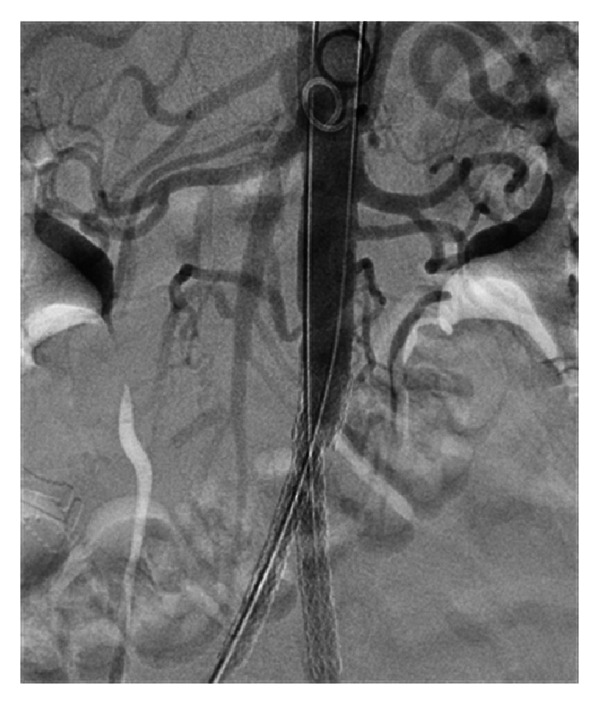
Reconstruction of the aortoiliac occlusion with an aortic stent and bilateral kissing iliac stents.

The patient was a 53-year old female with multiple cardiovascular factors, including hypertension, hypercholesterolemia, diabetes mellitus, coronary artery disease, and history of heavy smoking (60 pack-years; quit 3 years ago), who presented with bilateral lower extremity intermittent claudication, right worse than left, forcing her to stop after 3 minutes of walking. On physical examination, her bilateral femoral, popliteal, dorsalis pedis and posterior tibialis pulses were not palpable. At rest, her ankle-brachial index (ABI) measured 0.57 on the right and 0.58 on the left. She underwent a computed tomographic angiography (CTA) that showed complete occlusion of the distal abdominal aorta, including the origin of the inferior mesenteric artery, as well as the bilateral common iliac arteries (CIAs), with reconstitution at the level of the iliac bifurcations. The diameter of the abdominal aorta was 12 mm at the proximal aspect of the occlusion and decreased to 9 mm at the level of the bifurcation, as seen on the preplanning CTA. The right external iliac artery (EIA) was 4 mm and the left EIA was 5 mm in diameter. The occluded right CIA was 6 mm and the occluded left CIA was 7 mm in diameter. The patient declined open surgery and opted for endovascular therapy. Left brachial arterial access was obtained under ultrasound guidance using a micropuncture access kit (Cook Medical, Bloomington, IN, USA). A 5 French sheath was placed and a 5 French pigtail flush catheter (Cook Medical, Bloomington, IN, USA) was advanced into the abdominal aorta. An aortogram was performed and showed complete occlusion of the distal infra-renal aorta and bilateral CIAs, with reconstitution at the iliac bifurcations (Figures 1 and 2). Bilateral retrograde common femoral artery (CFA) access was obtained using ultrasound guidance and a micropuncture access kit. Five French sheaths were inserted in the CFA bilaterally and upsized later to an 8 French sheath on the left and 7 French on the right. Unfractionated heparin was injected intravenously in boluses throughout the procedure to maintain the activated clotting time (ACT) >250 seconds. A 5 French Davis catheter (Cook, Bloomington, IN, USA) and 0.035′′ Glidewire (Terumo, Somerset, NJ, USA) were used to perform antegrade subintimal recanalization of the right CIA from a brachial approach. The Glidewire was retrieved from the right femoral sheath (Figure 3) and then exchanged for a Rosen wire (Cook, Bloomington, IN, USA). Subintimal recanalization of the left CIA from a femoral approach was unsuccessful, with failure to reenter the true aortic lumen. Attempts to recanalize the left CIA from a brachial approach were also unsuccessful, as the wire and catheter continued to advance into the recanalized right CIA, following the path of the least resistance. To overcome this problem, the recanalized right CIA was occluded at its origin with a 5 mm × 40 mm UTD balloon catheter (Boston Scientific, Natick, MA, USA) inserted from the right femoral approach, sealing the origin of the right CIA. The 5 French Davis catheter was advanced from the brachial approach into the distal abdominal aorta to the level of the occluded left CIA. With the tip of the catheter pointed toward the occluded left CIA, the stiff back end of a 0.035′′ Glidewire was advanced to initiate a sharp recanalization of the occluded left CIA (Figure 4). The presence of the occlusion balloon prevented the Davis catheter from advancing into the recanalized right CIA and forced it in the direction of the occluded left CIA. The catheter was advanced over the wire into the occluded left CIA. At this point, the floppy end of the Glidewire was advanced through the catheter, and recanalization of the left CIA continued from a brachial approach. The wire reentered the true lumen of the left EIA and was retrieved from the left femoral sheath (Figure 5). The Glidewire was exchanged for a Rosen wire advanced into the abdominal aorta. From the left femoral approach, a 16 mm length Intrastent Doublestrut LD balloon expandable stent (EV3, Plymouth, MN, USA) mounted on 10 mm × 2 cm EverCross balloon catheter (EV3, Plymouth, MN, USA) was deployed in the proximal occluded aortic segment, and then further balloon dilated to 12 mm using a 12 mm × 2 cm EverCross balloon catheter (EV3, Plymouth, MN, USA) (Figure 6). More distally, the occluded aortoiliac bifurcation was reconstructed using a 6 mm × 59 mm iCAST balloon expandable covered stent (Atrium, Hudson, NH, USA) on the right and a 7 mm × 59 mm iCAST balloon expandable covered stent on the left, deployed simultaneously in a kissing fashion; the iliac stents were extended further to cover the entire length of the occluded common iliac arteries, ending just above the iliac bifurcations. Final arteriogram showed reestablishment of flow through the reconstructed aortoiliac bifurcation with no significant residual stenosis (Figure 7). The patient was discharged the next day on Clopidogrel 75 mg once a day for 3 months and aspirin 325 mg once a day for life. She noted significant clinical improvement with an ABI of 0.85 on the right and 1.01 on the left at her 2-week postprocedure followup. She was seen in followup at 6 months, with recurrence of intermittent claudication on the right after 15 minutes of walking. Her right ABI decreased to 0.69; her left ABI was stable and measured 1.05. A CTA showed severe stenosis at the proximal aspect of the right CIA stent. Her symptoms were subjectively described as mild and nondisabling and she declined any further intervention. 

## 3. Discussion

The standard of care in patients with total aortoiliac occlusion is surgical and includes endarterectomy, graft insertion, and anatomic or extra-anatomic bypass, all considered major interventions [1, 3, 5]. Consequently, these patients are often denied surgery and remain untreated for a long time.

In a small series of 11 patients with total aortoiliac occlusion, treated with endovascular reconstruction, bilateral success was achieved in 8 patients (73%). Unilateral success, with recanalization of only one iliac artery, was achieved in the remaining 3 patients (27%). At the median followup of 14 months, all patients were alive and reported marked relief of symptoms with improvement in clinical status by at least one Rutherford category. Pending long-term outcomes and experience with a greater number of patients, the endovascular approach holds promise to replace surgery as the first line of treatment in patients with extensive aortoiliac disease [1]. Percutaneous Intentional Extraluminal Recanalization (PIER) can be safely performed in the iliac territory. The reentry level from a femoral retrograde approach into the aorta can be unpredictable, however, and the risk of extending the subintimal dissection into a patent aortic segment is real. This situation has been described as one of the most common reasons for failure [6]. Therefore, many interventionalists prefer the brachial approach [7]. Once one of the occluded iliac arteries is recanalized, crossing the contralateral iliac occlusion from a brachial approach can become more challenging, as the wire and catheter will follow the path of the least resistance into the recanalized iliac segment. A guiding sheath can provide support for the wire and catheter during recanalization; in the setting of flush CIA occlusion or aortoiliac occlusion, without a stump at the origin of the CIA, the guiding sheath can have difficulty engaging with the lesion, and the advantage of using the stiff support of the guiding sheath is negated [8]. The crossover recanalization approach is another alternative, where a curved diagnostic catheter, advanced from a contralateral femoral approach, is positioned at the aortic bifurcation with the tip engaging the stump of the iliac occlusion. A hydrophilic angled-tip Glidewire (Terumo, Somerset, NJ, USA) is used to cross the iliac occlusion and externalized through a sheath placed in the CFA ipsilateral to the treated CIA. Again, this technique is limited in flush iliac and complete aortoiliac occlusions, as by definition there is no iliac stump to engage with [9].

A curved sheathed needle can be used to puncture into the true lumen of the aorta, from the subintimal tract during recanalization of the CIA from an ipsilateral retrograde approach [10]. In brief, the technique consists of placing an occlusion balloon in the true lumen of the distal abdominal aorta advanced through the patent contralateral iliac system using a femoral approach. An 18-guage curved sheathed-needle is advanced through the ipsilateral subintimal tract of the iliac artery: the needle is sheathed at this point and used to indent the occlusion balloon and then advanced further rupturing the balloon. A wire is advanced through the needle once the position in the true lumen of the distal aorta is confirmed [11]. 

Re-entry into the true lumen can be facilitated by reentry devices like the Outback LTD catheter (Cordis, Miami Lake, FL, USA) and Pioneer catheter (Medtronic Cardiovascular, Santa Rosa, CA, USA). The initial experience primarily obtained in the femoral arteries supports their clinical value. Experience with these technologies in the iliac arteries is still somewhat limited; however, a few small series reported high technical success rate with these devices in the aortoiliac territory [12, 13]. 

A recently published technical note described recanalization of a flush iliac occlusion with the assistance of a contralateral iliac occlusive balloon in a patient with a chronic total occlusion of the left CIA; the distal abdominal aorta and right CIA were patent [8]. We used a very similar technique: our patient; however, had a total aortoiliac occlusion. 

## 4. Conclusion

Endovascular recanalization of a total aortoiliac occlusion is technically challenging, with the inability to reenter the true lumen at the level of the aorta, being one of the most common causes of failure, when attempted from a femoral approach. A brachial approach can offer some advantage, but once an occluded iliac artery is recanalized, crossing the contralateral iliac occlusion could become more challenging, as the wire and catheter tend to follow the path of least resistance to the recanalized iliac artery. Placement of a contralateral occlusion balloon could assist in recanalizing the other iliac occlusion. The real value of this technique is yet to be determined in a larger series with reproducible results. 

## Abbreviations

CIA: Common iliac artery
EIA: External iliac artery
CFA: Common femoral artery
ACT: Activated clotting time
TASC: Trans-Atlantic Inter-Society Consensus
ABI: Ankle-brachial index
CTA: Computed tomographic angiography.
